# Regimen

**Published:** 1868-08

**Authors:** 


### REGIMEN.

RIGHT well hath some old codger of the ancient time written in respect to health and its preservation; doubtful are we that any man of this diluting age could possibly comprise so much sound sense in as few words as those which follow:

There is a wisdom in this beyond the rules of physic. A man's own observation what he finds good of, and what he finds hurt of, is the best physic to preserve health; but it is a safer conclusion to say: "This agreeth not well with me, therefore I will not continue it;" than this—"I find no offense of this, therefore I may use it;" for strength of youth in nature passeth over many excesses which are owing a man till his age. Discern of the coming on of years, and think not to do the same things still; for age will not be defied. Beware of sudden change in any great point of diet; and if necessary, enforce it, fit the rest to it; for it is a secret both of nature and state, that it is safer to change many things than one. Examine thy customs of diet, sleep, exercise, apparel, and the like; and try in any thing thou shalt judge hurtful, to discontinue it little by little; but so, as if thou dost find any inconvenience by the change thou come back to it again; for it is hard to distinguish that which is generally held good and wholesome from that which is good particularly, and fit for thine own body. To be free-minded and cheerfully disposed at hours of meat, and of sleep, and of exercise, is one of the best precepts for long lasting. As for the passions and studies of the mind, avoid envy, envious fears, anger, fretting inwards, subtle and knotty inquisitions, joy and exhilarations in excess, sadness not communicated. Entertain hopes; mirth rather than joy; variety of delights rather than surfeit of them; wonder and admiration, and therefore novelties; studies that fill the mind with splendid and illustrious objects—as histories, fables and contemplations of nature.

